# Use of alternative medicine, ginger and licorice among Danish pregnant women – a prospective cohort study

**DOI:** 10.1186/s12906-018-2419-y

**Published:** 2019-01-05

**Authors:** Tabia Volqvartz, Anna Louise Vestergaard, Sissel Kramer Aagaard, Mette Findal Andreasen, Iana Lesnikova, Niels Uldbjerg, Agnete Larsen, Pinar Bor

**Affiliations:** 1 Department of Obstetrics and Gynecology, Randers Regional Hospital, Randers, Denmark; 2 Department of Biomedicine, Pharmacology, Aarhus University, Aarhus, Denmark; 3 Section for Forensic Chemistry, Department of Forensic Medicine, Aarhus University, Aarhus, Denmark; 4 Department of Pathology, Vidant Medical Center, Greenville, North Carolina USA; 5 Department of Obstetrics and Gynecology, Aarhus University Hospital, Aarhus, Denmark

**Keywords:** Alternative medicine, First trimester pregnancy, Ginger, Herbal medicine, Licorice

## Abstract

**Background:**

The use of alternative medicines and dietary supplements is constantly changing, as are dietary habits. One example of this phenomenon is the current popularity of ginger products as an everyday health boost. Ginger and licorice has also been shown to ameliorate nausea a common complaint in early pregnancy. Alternative medicines are often regarded as safe. However, they might affect fetal development, such as through alterations of hormone metabolism and cytochrome P450 function. Health care professionals may be unaware of the supplementation habits of pregnant women, which may allow adverse exposures to go unnoticed, especially if the rates of use in pregnancy are not known. We therefore investigated the use of alternative medicines and licorice among pregnant Danish women.

**Methods:**

A total of 225 pregnant women were included in a prospective cohort when attending the national prenatal screening program at gestational weeks 10–16. Participants were asked to complete a questionnaire regarding their socio-economic status and lifestyle habits, including their intake of alternative medicine and licorice.

**Results:**

We found that 22.7% of women reported taking alternative medicines, with 14.7% reporting daily consumption. Ginger supplements were consumed by 11.1%, mainly as health boost and 87.1% reported consumption of licorice. Regular or daily licorice consumption was reported by 38.2 and 7.1%, respectively. Notably, the use of licorice was reflected by an increase in blood pressure of the pregnant women.

**Conclusions:**

The use of licorice and alternative medicines appears to be common in pregnant Danish women, supporting the need for further investigations into the safety of alternative medicine use during pregnancy and the importance of up-to-date personalized counseling regarding popular health trends and lifestyle habits.

**Electronic supplementary material:**

The online version of this article (10.1186/s12906-018-2419-y) contains supplementary material, which is available to authorized users.

## Background

In modern society, everyday lifestyles are constantly changing. With the plethora of internet-based platforms, new health trends can spread rapidly among pregnant women. Such habits may escape the attention of health care professionals, allowing adverse exposures in early pregnancy to go undetected. International data supports the hypothesis that pregnant women perceive herbal and conventional medications as quite harmless [1]. Moreover, a recent study reported that recommendations to take contra-indicated herbal medicines during pregnancy may come directly from health care professionals [2].

A multinational study from 2016 suggested that up to 60% of all pregnant women use herbal-based alternative medicines [2]. However, cultural differences in the use of alternative medicine are well-established, even within the Scandinavian and Nordic countries. In Iceland, the prevalence has been reported to be as high as 35%, in contrast to Norway and Sweden where the prevalence has been reported to be 17 and 4%, respectively [3]. However, the prevalence of such use among pregnant women in Denmark has not previously been published.

Importantly, the safety of many herbal remedies has never been investigated in human pregnancies, as no strict rules for safety testing apply to alternative medicine [4] despite their teratogenic potential [5]. Several adverse effects caused by alternative medicine in pregnancy have been described, including miscarriage, preterm delivery, and malformations. Interestingly, recent data suggest that between 2.5–13% of pregnant women use alternative medicines together with prescribed medications [6]. However, there are limited published data investigating the adverse effects of alternative medicine due to their direct chemical toxicity, herbal-drug or herbal-herbal interactions.

Studies have shown that the majority (76%) of women who self-administer herbal medicine during pregnancy do not disclose their use to their doctor or midwife [7], making such use a potential safety concern.

Common exposure during pregnancy includes the use of ginger, which for decades has been the most widely used herbal remedy in the management of pregnancy-related nausea and vomiting. In addition, ginger is thought to strengthen the immune system and generally boost human health, leading to an increased popularity in recent years. At present, ginger is often added to consumables routinely sold in Danish supermarkets, such as teas and shots. The use of ginger as a health booster could lead to increased and continuous consumption throughout pregnancy, yet no guidelines currently exist in relation to the permissible amount of ginger exposure, even though ginger as a dietary supplement is not recommended by the Danish Veterinary and Food Administration. Several studies have found occasional use of ginger as an anti-emetic to be safe [8]. However, recent reports have shown that pharmacologically active substances in ginger may increase the risk of bleeding by decreasing platelet aggregation [9], and ginger-based compounds have been suspected to increase the risk of stillbirth [10]. If unaware of these potential risk pregnant women might continue their ginger habits throughout pregnancy – in particular if also obtaining a reduction of nausea that might give them the impression that ginger is well-tolerated by the body. Originally, licorice was used against upper respiratory problems and stomach inflammation while today it is primarily eaten for pleasure. However, a double-blind randomized study found a positive effect of licorice in prevention of acid reflux and vomiting [11]. Nausea and vomiting are common complains during pregnancy underlining that eating habits may also reflect a subconscious “self-medication” strategy in particular in Scandinavia were licorice based candies are well-known and popular. However, licorice can increase blood pressure, and the content of glycyrrhizin - the major active constituents of licorice - can also decrease platelet aggregation [12] making it a key problem that several candy products with licorice is increasingly being consumed during pregnancy.

As a systematic report of the current use of licorice, alternative medicines and other herbal supplements among pregnant Danish women has never been reported, the aim of this study was to assess the prevalence and characteristics of alternative medicine, ginger and licorice use among Danish pregnant women.

## Methods

In this prospective cohort study, we included participants seen at the Department of Obstetrics and Gynecology, Randers Regional Hospital, Denmark between June 2016 and December 2016. The inclusion criteria was attendance at a routine ultrasound examination in gestational week 10–16, which is accepted by more than 95% of the pregnant women in the recruitment area where this scan is not offered elsewhere. Exclusion criteria included an age below 18 years and poor language skills.

The study was approved by the Regional Scientific Ethical Committee (VEK 1–10–72-75-16) and followed the Helsinki guidelines of informed consent. Due to additional obligations, we could only recruit participants 2–3 days a week. On these days all eligible women were invited in correlation with their random given times at the ultrasound unit (Table 1).

**Table 1:** Demographic characteristics of the cohort

Demography	% (n)
Maternal age (years)	
20–29	50.9 (114)
30–39	44.6 (100)
≥ 40	4.5 (10)
Parity	
Nulliparous	41.5 (93)
Primiparous	44.6 (100)
Multiparous	13.8 (31)
Maternal pre-pregnancy BMI (kg/m2)	
Underweight (< 18,5)	1.8 (4)
Normal (18,5–24,9)	43.3 (97)
Overweight (25–30)	29.9 (67)
Obese (> 30)	25.0 (56)
Chronic health issues	28.6 (64)^a^
No chronic health issue	71.4 (160)
Married	42.0 (94)
In relationship, cohabitating	54.0 (121)
In relationship, non-cohabitating	2.2 (5)
Single	1.3 (3)
Other	0.4 (1)
Highest completed education level	
Elementary school	3.6 (8)
Upper secondary school	7.6 (17)
Vocational education	25.0 (56)
Shorter level of education	12.9 (29)
Bachelor degree	37.9 (85)
Master degree	12.1 (27)
Other	0.9 (2)
Household income	
< 103,000 DKK	0.9 (2)
103,000–200,000 DKK	3.6 (8)
200,000–500,000 DKK	35.3 (79)
500,000–800,000 DKK	46.4 (104)
> 800,000 DKK	13.8 (31)

^a^Chronic health issues were self-reported, such as asthma and allergies, metabolic disorders, dermatitis, cardiovascular disorders, bowel disorders, autoimmune disorders and previous cancers

The participants answered a questionnaire at recruitment or during a phone call within 1–2 weeks regarding the following information: age, parity, Body Mass Index (BMI), smoking, alcohol, licorice intake, socio-economic status, educational level, use of prescription and over the counter medicine (OTC), supplemental vitamins, and intake of alternative medications including herbal supplements (e.g., teas). If the women used any health supplements or followed a specific nutritional lifestyle, they were asked to specify the amount and duration of the various intakes. After delivery, we obtained information regarding outcomes from the women’s electronic medical records (Additional file 1: Table S2).

### Statistics

Continuous variables were compared between groups using Student’s tests or Mann-Whitney testing based on the testing of normal distribution of the data. Two-tailed comparisons were performed unless otherwise noted. Data are summarized as the means ± SD. GraphPad Prism version 7.03 Software (GraphPad Software, Inc., San Diego, CA, USA) was used to analyse the Student’s t-test and Mann-Whitney test results as well as confidence intervals presented in the relevant Tables. A level of significance at or below 0.05 was considered statistically significant for all analyses.

## Results

Among 297 eligible Danish women attending a routine first trimester ultrasound scan during our presence, we included 225 (75.8%) women who accepted to participate in the study, corresponding to a prevalence of 23.1% of all birth at Randers Regional Hospital in the inclusion period. Lack of time was the most common reason given for non-participation.

All women were interviewed, all but one returned the completed questionnaire living a study population for this study of 224.

The vast majority 71.8% (*n* = 158) had a spontaneous vaginal delivery (for details on birth complications and new born Apgar score see Additional file 1: Table S2). Of the 224 women from whom questionnaire data were available, data on birth weight and infant health was available from 217 participants. Of the seven missing individuals, four had a spontaneous abortion (1.8%), and three (1.3%) were lost to follow up at birth, because they had moved to another district (Additional file 2: Figure S1). Information regarding the correspondence between exposures and outcome was based on the 217 women that gave birth at Randers Regional Hospital. (For details on parity and lifestyle see Additional file 3: Table S1).

Up to 22.7% (*n* = 51) reported consumption of at least one type of alternative medicine, 14.7% (*n* = 33) reported doing so daily, and 4.9% (*n* = 11) took more than one remedy regularly. This intake was associated with chronic health issues (31.3%; 20 of 64), of which the majority reported a daily use (20.0%; 13 of 65), and a high household income (32.3%; 10 of 31; Table 2).

**Table 2:** Household income, educational level and nutritional habits

Household income and educational level	Total number	Alternative medicines and herbal remedies % (n)	Ginger consumption 1st trimester % (n)	Licorice intake 1st trimester % (n)
Income in Danish Kroner				
< 103,000–200,000 DKK	10	0.0 (0)	0.0 (0)	80.0 (8)
200,000–500,000 DKK	79	24.1 (19)	10.1 (8)	84.8 (67)
500,000–800,000 DKK	104	21.2 (22)	11.5 (12)	91.3 (95)
> 800,000 DKK	31	32.3 (10)	16.1 (5)	83.9 (26)
Educational level				
Elementary school	8	12.5 (1)	12.5 (1)	87.5 (7)
Upper secondary school	17	17.6 (3)	5.9 (1)	88.2 (15)
Vocational education	56	21.4 (12)	7.1 (4)	82.1 (46)
Shorter level of education	29	20.7 (6)	13.8 (4)	89.7 (26)
Bachelor degree	85	24.7 (21)	12.9 (11)	91.8 (78)
Master degree	27	25.9 (7)	14.8 (4)	81.5 (22)

Two participants were not included in educational level; one did not have an educational diploma, and one had an educational level not represented by the educational categories

Summary of the socioeconomic status among the women and their use of alternative medicines and herbal remedies with focus on ginger and licorice

Ginger products were the most frequently used form of alternative medicine (11.1%, *n* = 25), such as through shots (7.1%; *n* = 16), tea, tablets, and oil. Among these, 3.1% (*n* = 7) reported taking them alongside prescription or OTC medicine. Only 2.7% (*n* = 6) used ginger for nausea and vomiting. The intake of ginger products was associated with chronic health issues (17.2%; 11 of 64), mean maternal age 31.8 years (95% CI: 29.9–33.6) among users vs. 29.5 years (95% CI: 28.6–30.1) among non-users (*p* = 0.02), mean birthweight 3572 g (95% CI: 3316-3827 g) among exposed vs. 3440 g (95% CI: 3355-3525 g) among unexposed (*p* = 0.28). Additionally, the consumption rate of ginger increased with higher levels of education, lowest among women with an upper secondary school education (5.9%; 1 of 17) and highest among women with a master’s degree (14.8%; 4 of 27).

Other frequently used products included peppermint tea for nausea (1.8%; *n* = 4), Psyllium Husk Fiber® for obstipation (6.7%; *n* = 15), and Kräuterblut® as an herbal substitute for iron supplements (2.7%; *n* = 6), see Table 3 and Table 4.

**Table 3:** Consumption of alternative medicine and herbal ailments

Intake of alternative medicine and supplements	% (n)	Possible risk in pregnancy / recommendations during pregnancy
Alternative medicines	22.7(51)	
Psyllium Husk Fiber	6.7(15)	Delayed absorption of other drugs, necessary insulin dosage adjustment (downward) for diabetics [30]
Valerian	0.4(1)	Influence on fetal ossification, cytotoxic and mutagen [5]
Glucosamines	0.4(1)	No available information
Ginger	11.1(25)	Induce abortion, influence fetal testosterone metabolism and maternal vaginal bleeding from gestational week 17 [5, 31]
Pregnancy tea (raspberry leaves and ginger)	0.4(1)	Antigonadotrophic effects [5]
Mint tea	1.8(4)	Emmenagogue properties [30]
Cranberry tablets	0.4(1)	Insufficient treatment of UVI [3]
Kefir	0.4(1)	Contains small amounts of alcohol (fermented)
Kombucha tea	0.4(1)	Contains small amounts of alcohol (fermented)
Krauterblüt (herbal iron remedy)	2.7(6)	Iron deficiency due to insufficient supplementation [32]
Thyme tea	0.4(1)	Inducing abortion [2]
L-lysine	0.4(1)	No available information
Green tea	0.4(1)	Contains caffeine [30]
Turmeric	0.4(1)	Induces abortion [33] and is cytotoxic [34]
Lactic Lactobacillus acidophilus bacteria	0.9(2)	No available information
Boldocynara (Boldo, dandelion, mint, artichoke)	0.4(1)	No available information, but mint has emmenagogue properties [30]
Oregano	0.4(1)	No available information
Essential (Norwegian remedy)	0.4(1)	No available information
MK oil (Linseed, evening primrose, rosehip, caraway)	0.4(1)	Caraway has emmenagogue properties and spasmolytic effects [30], evening primrose increases the incidence of prolonged rupture of membranes, oxytocin augmentation and vacuum extraction [31]

Overview of the reported use of alternative medicines by the women and an outline of the current recommendation and reported risk aspects in pregnancy from the medical literature

**Table 4:** Usages of alternative medicines

Name *Scientific name*	Ethnomedical use	Verified scientific use	Use in Danish setting
Artichoke, in Boldocynara *Cynara scolymus*	Loss of appetite, dyspeptic complains, and prophylactic against reemission of gallstones and as a tonic in convalescence [30]	No data of clinical efficacy^a^	Against flatulence^c^
Boldo, in Boldocynara *Peumus boldus*	Dyspeptic complaints [30]	No data of clinical efficacy^a^	Against flatulence^c^
Brown Kelp, in Krauterblüt® *Macrocystis pyrifera*	Weight reduction, against hypertension and anemia in pregnancy [30]	No clinical data	Ensure a good supply of iron, riboflavin, pyridoxines, cobalamin and vitamin C^c^
Caraway, in MK oil *Carum carvi*	Gastrointestinal cramps, flatulence, feelings of fullness, improve lactation, an emmenagogue [30]	Insufficient data of clinical efficacy^a^	Anti-ageing properties with vital vitamins, minerals and bioflavonoids^c^
Cascarilla, in Krauterblüt® *Croton eluteria*	Digestive disorders, diarrhea and vomiting [30]	No clinical data	Ensure a good supply of iron, riboflavin, pyridoxines, cobalamin and vitamin C^c^
Cranberry *Vaccinium macrocarpon*	Against urinary tract irritation, gout, rheumatism and calculus [30]	Mixed data of clinical efficacy [35]	Prevention and treatment of lighter, recurrent urinary tract infections^b^
Dandelion, in Boldocynara *Taraxacum officinale*	Acute mastitis, urinary disorders, chronic ulcers, tuberculosis, flatulence, colic, kidney disease, gout, jaundice and biliary stones [30]	No data of clinical efficacy^a^	Against flatulence^c^
Evening primrose, in MK oil *Oenothera biennis*	Neurodermatitis, premenstrual syndrome, dietary aid, high cholesterol levels, menopausal hot flashes, mastalgia and treatment of hyperactivity in children [30]	Insufficient data of clinical efficacy^a^	Anti-ageing properties with vital vitamins, minerals and bioflavonoids^c^
Fennel, in Krauterblüt® *Foeniculum vulgare*	Peptic discomforts, disorders of the gastrointestinal tract, feeling of fullness, flatulence and catarrh of the upper respiratory tract [30]	Clinical data limited. Pharmacological data supports the use against mild spasmodic gastro-intestinal complaints, menstrual cramps and expectorants in cough with colds^a^	Ensure a good supply of iron, riboflavin, pyridoxines, cobalamin and vitamin C^c^
Field Horsetail, in Krauterblüt® *Equisetum arvense*	Tuberculosis, catarrh in the kidney and bladder regions, a hematostatic for profuse menstruation, nasal, pulmonary and gastric hemorrhages, rheumatic diseases, gout, poorly healing wounds, swelling, fractures, frostbite and loss of hair [30]	Insufficient data of clinical efficacy^a^	Ensure a good supply of iron, riboflavin, pyridoxines, cobalamin and vitamin C^c^
Garden angelica, in Krauterblüt® *Angelica archangelica*	Used against loss of appetite, dyspeptic and menstruation complaints, liver and biliary duct conditions, coughs and bronchitis [30]	Insufficient data of clinical efficacy. However, coumarin-derivatives in *Angelica archangelica* are phototoxic^a^	Ensure a good supply of iron, riboflavin, pyridoxines, cobalamin and vitamin C^c^
Ginger *Zingiber officinale*	Used as a carminative, expectorant, and astringent. To treat colds, shortness of breath, nausea, vomiting, dyspeptic symptoms and pharyngitis [30]	Antiemetic^a^	No available data
Glucosamines	Originally used in veterinary medicine	Limited data	Prevention and treatment of osteoarthritis^c^
Green tea *Camellia sinensis*	Stomach disorders, nausea, migraine, symptoms of fatigue, vomiting, diarrhea, cardiac and circulatory conditions, states of agitation, states of depression, pain, fever and fatigue [30]	Stimulatory effect^a^ (corroborated with the caffeine content)	No available data
Hibiscus, in Krauterblüt® *Hibiscus sabdariffa*	Diuretic, mild laxative, treatment of hypertension, pyrexia, cough, colds, malaria and skin inflammations [36]	Insufficient data of antihypertensive efficacy [37]	Ensure a good supply of iron, riboflavin, pyridoxines, cobalamin and vitamin C^c^
Kefir (fermented probiotic milk product)	Limited data	Limited data	No available data
Kombucha tea	Weight loss and anticancer properties [38]	No clinical data	No available data
Lactic Lactobacillus acidophilus bacteria (fermented probiotic milk product)	Introduced in the early twentieth century. To normalize the bacterial flora in the gut.	Mixed data of clinical efficacy [39]	Normalization of the intestinal flora^b^
Licorice *Glycyrrhiza glabra*	Sore throats, appendicitis, constipation, and to increase milk production and micturition [30]	Insufficient data of clinical efficacy^a^	Mucous release effect for cough in shorter periods^b^
Linseed, in MK oil *Linum usitatissimum*	For coughs, bronchial conditions, urethritis, diarrhea and gonorrhea [30]	Mixed data of clinical efficacy. However, effectiveness of treatment of habitual constipation and softening of stool is plausible^a^	Anti-ageing properties with vital vitamins, minerals and bioflavonoids^c^
L-lysine	Limited data	Limited data	
Mint *Mentha piperita*	Nausea, vomiting, morning sickness, respiratory infections, dysmenorrhea and colds [30]	Symptomatic relief of digestive disorders^a^	Indigestion and flatulence^b^
Nettle, in Krauterblüt® *Urtica dioica*	Hematogenic, rheumatic remedy, diuretic [30]. For diabetes, hypertension and prostate cancer [40]	Insufficient data of clinical efficacy^a^	Ensure a good supply of iron, riboflavin, pyridoxines, cobalamin and vitamin C^c^
Oregano *Origanum vulgare*	Colds, fever, cough, vomiting, dyspepsia painful menstruation, rheumatoid arthritis, urinary tract disorders, dysentery, jaundice and malnutrition for children [30]	Limited data	No available data
Physillium Husk Fibre *Plantago ovata*	Against inflammation of the mucous membrane in the urogenital and gastrointestinal tract [30]	Treatment of habitual constipation, desirable soft stool and adjuvant to diet in hypercholesterolemia^a^	Against constipation, diarrhea, to increase fiber intake and adjuvant to diet in hypercholesteremia^b^
Quackgrass, in Krauterblüt® *Agropyron repens*	Urinary tract infections [30]	No data of clinical efficacy^a^	Ensure a good supply of iron, riboflavin, pyridoxines, cobalamin and vitamin C^c^
Raspberry leaves*Rubus idaeus*	Relieve nausea and induce labour [30]	Insufficient data of clinical efficacy^a^	No available data
Rosehip, in MK oil*Rosa rugosa*	Disorders in the efferent urinary tract, the kidneys, kidney stones, rheumatic conditions, gout, colds, scurvy, febrile conditions [30]	Limited data	Anti-ageing properties with vital vitamins, minerals and bioflavonoids^c^
Spinach, in Krauterblüt® *Spinacia oleracea*	Ailments of the gastrointestinal tract and blood generating [30]	Limited data	Ensure a good supply of iron, riboflavin, pyridoxines, cobalamin and vitamin C^c^
Thyme *Thymus vulgaris*	Catarrh of the upper respiratory tract, asthma, laryngitis, cough, gastritis and dyspepsia [30]	No data of clinical efficacy^a^	Expectorate by productive cough in combination with *Primula veris*^b^
Turmeric *Curcuma longa*	Dyspeptic disorders and inflammations [30]	No data of clinical efficacy^a^	No available data
Yarrow, in Krauterblüt® *Achillea millefolium*	Laxative and treatments against bleeding hemorrhoids, menstrual complaints and gynecological agents [30]	No data of clinical efficacy^a^	Ensure a good supply of iron, riboflavin, pyridoxines, cobalamin and vitamin C^c^
Wormwood, in Krauterblüt® *Artemisia absinthium*	Ailments of gastrointestinal tract, liver disorders, bloating, anemia, irregular menstruation, intermittent fever, loss of appetite and worm infestation [30]	No data of clinical efficacy^a^	Ensure a good supply of iron, riboflavin, pyridoxines, cobalamin and vitamin C^c^

^a^According to scientific rapports by European Medicines Agency (EMA)

^b^Approved by the Danish Medicines Agency

Please note that in a Danish context an herbal remedy can only be sold if approved by the authorities. However, this approval does not demand any scientific evidence and all though the remedies can only be advertised according to their approval they are sold freely and may be used for other purposes

^c^Not approved as an herbal remedy. Therefore, stating manufacturers own clarification of remedy on the Danish market

Overview of the common ethnomedical indications and possible scientific verified use concerning the reported use of alternative medicines and their potential indication for use in a Danish setting

**Fig. 1:**
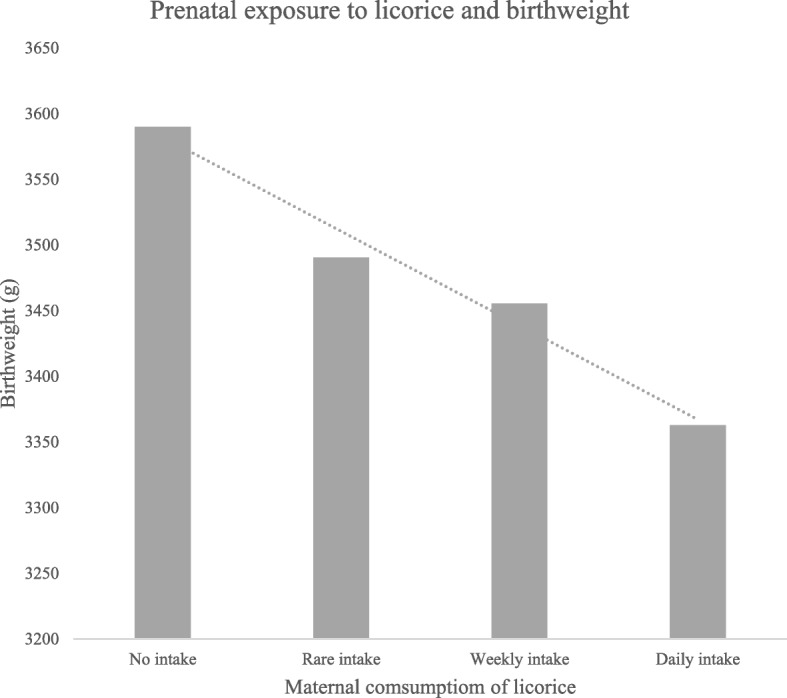
Association between birthweight and prenatal exposure to maternal licorice consumption. Maternal consumption of licorice showed an association with a reduced birthweight. Mean birthweight 3590 g, 95% CI: 3327-3853 g in the group with no intake^A^, mean birthweight 3490 g, 95% CI: 3392-3589 g (*p* = 0.62) in the group with a rare intake, mean birthweight 3455 g, 95% CI: 3335-3575 g (*p* = 0.35) in the group with a weekly intake vs. mean birthweight 3363 g, 95% CI: 3010-3716 g (*p* = 0.29) in the group with a daily intake. ^A^Reference group

No less than 87.1% (*n* = 196) reported consuming licorice, 38.2% (*n* = 86) reported licorice consumption at least “a couple of times a week,” and 7.1% (*n* = 16) reported daily use. On each occasion of consumption, 33.8% (*n* = 76) ate at least a handful of licorice candies, and 4.4% (*n* = 10) reported consuming an entire bag of licorice. All participants with hypertension (1.3%; *n* = 3) reported a weekly consumption of licorice. Moreover, the frequency of licorice intake was also associated, albeit not significantly, with reduced birthweight (see Fig. 1). Differences in mean blood pressures between women reporting a mean daily intake of licorice (123 mmHg, 95% CI: 116–130 mmHg) and women reporting rare or no intake of licorice (119 mmHg, 95% CI: 117–121 mmHg) were significantly associated with increased maternal systolic blood pressure (*p* = 0.04), estimated via one-tailed Mann-Whitney testing based on the known effects of licorice on blood pressure (see Table 5).

**Table 5:** Maternal blood pressure at gestational week 29 in relation to licorice consumption

Licorice consumption	BMI Mean, 95% CI	Systolic blood pressure mmHg Mean, 95% CI	Diastolic blood pressure mmHg Mean, 95% CI	*P*-value systolic/diastolic
Daily	27.44 (23.91–30.97)	122.81 (116.01–129.61)	75.06 (69.86–80.26)	0.04/0.51^b^
Weekly	27.41 (26.04–28.77)	119.37 (116.62–122.12)	75.35 (73.53–77.18)	0.34/0.42^b^
Rarely^a^	25.81 (24.81-26.81)	118.82 (116.37–121.27)	75.82 (74.10–77.53)	
Never^a^	27.72 (25.18-30.26)	118.19 (113.77–122.60)	72.93 (68.08–77.77)	

^a^Reference groups

^b^One-tailed Mann-Whitney test

Among the women reporting daily consumption of licorice in first trimester, the measured blood pressure later in pregnancy was significant higher than the women who reported a rarely or no intake

## Discussion

The main finding of this cohort study of Danish pregnant women was that 23% reported using alternative medicines, with ginger products being by far the most popular item. Furthermore, 87% reported consuming licorice, with 7% reporting daily use.

Performing the study in an unbiased manner where the eligible population was representative of the entire population in the geographic area, and achieving an overall participation rate of 76%, strengthen the value and validity of the findings. Notably, we cannot exclude that selection bias occurred, as the women had to accept participation. However, ultra-performance liquid chromatography with high-resolution time-of-flight mass spectrometry (UPLC-HR-TOFMS) analysis of the pharmacological content of the blood samples from the same cohort (Volqvartz and Vestergaard et al. in prep) indicates a high degree of consistence between results from this cohort and a similar UPLC-HR-TOFMS analysis of unselected, unbiased pregnant women from the same region performed by Aagaard and co-workers [13]. Also, the organisation of the maternal care system supports that all women from all parts of society attends the same, free-of-charge prenatal diagnostic system. On the other hand, limiting our study to one single interview means we did not assess all exposures occurring in pregnancy and, the size of the cohort limits the power to demonstrate possible associations between exposure and adverse obstetric outcomes in particular if the exposures are rare. However, by asking the women specifically if ginger or other substances were used do to pregnancy related nausea or for other reasons we obtained indication of the duration of the use.

Finally, we did not specifically inquire regarding the intake of red-clover-containing pregnancy tonics, which might explain why this was not mentioned by any participant. Notably, red clover is rich in phytoestrogens [14], and prenatal exposure is suspected to have deleterious effects on the developing male reproductive system; knowledge of the red-clover use among our subjects would thus be valuable. Analysis of 7928 boys in the cohort of “The Avon Longitudinal Study of Parents and Children” (ALSPAC) found that a maternal vegetarian diet rich in phytoestrogens in pregnancy was associated with an increased risk of hypospadias [15].

The fraction (23%) of pregnant women taking alternative medicines in this Danish study is relatively high compared to previous studies in Sweden (4%), Finland (9%), Norway (17%), and Iceland (35%) [3], but not compared to a number of non-Nordic countries (29%) [3]. However, the fraction appears lower than that among non-pregnant Danish pre-surgical patients of both genders (50%) [16]. The comparison of these studies is limited to some extent by different study designs and different definitions of alternative medicine. However, the positive associations with household income and education level is consistent across several studies [1]. With the size of this study, assessing specific adverse effects on fetal development might not be possible even with our focus on the time of organogenesis. However, studies of the Danish National Birth Cohort have found a higher risk for malformations after prenatal exposure to St. John’s wort [17]. This herbal medicine is a “natural anti-depressant” known to affect the serotonin system in ways similar to conventional antidepressants, which is a potential safety concern in pregnancy. With the high prevalence of users of alternative medicines identified in this study, further studies into the teratogenic effects of other exposures are warranted.

**Fig. 2:**
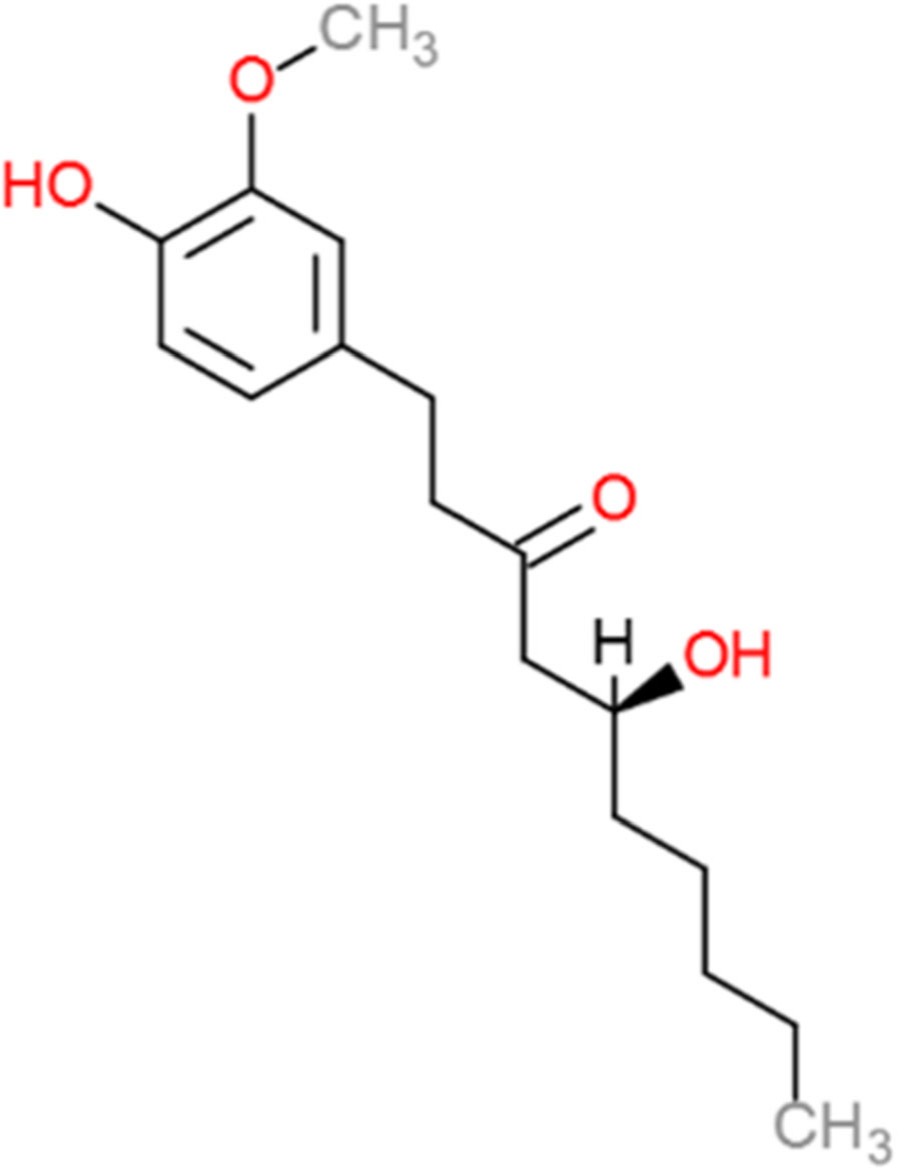
The chemical structure of 6-gingerols, the main active constituent of ginger, *Zingiber officinale*. Modified after Qui et al. [19]. 6-gingerol is derived from the phenylalanine pathway and has potential to be anti-septic, to have anti-cancer properties and reducing nausea and migraine

In particular, it appears important to focus on the use of ginger products among pregnant women. In Denmark, supermarkets expect to double the selling of ginger shots in coming years [18], even though 77% of the ginger consumers in our study did not declare their intake to be caused by a need to relieve symptoms. Ginger, or its active compound 6-gingerol (see Fig. 2) interacts with the cytochrome P450 system (e.g., CYP3A4, CYP2C9) [19] and fetal testosterone metabolism [20], thus serving as a potential teratogenic item. Furthermore, a cohort study from Korea showed 4 stillbirths among 159 singleton-pregnancy women receiving dried ginger (OR = 7.8; 95% CI 2.9–21) compared to the general population [10]. In addition, ginger decreases platelet aggregation, which may increase the risk of post-partum bleeding.

**Fig. 3:**
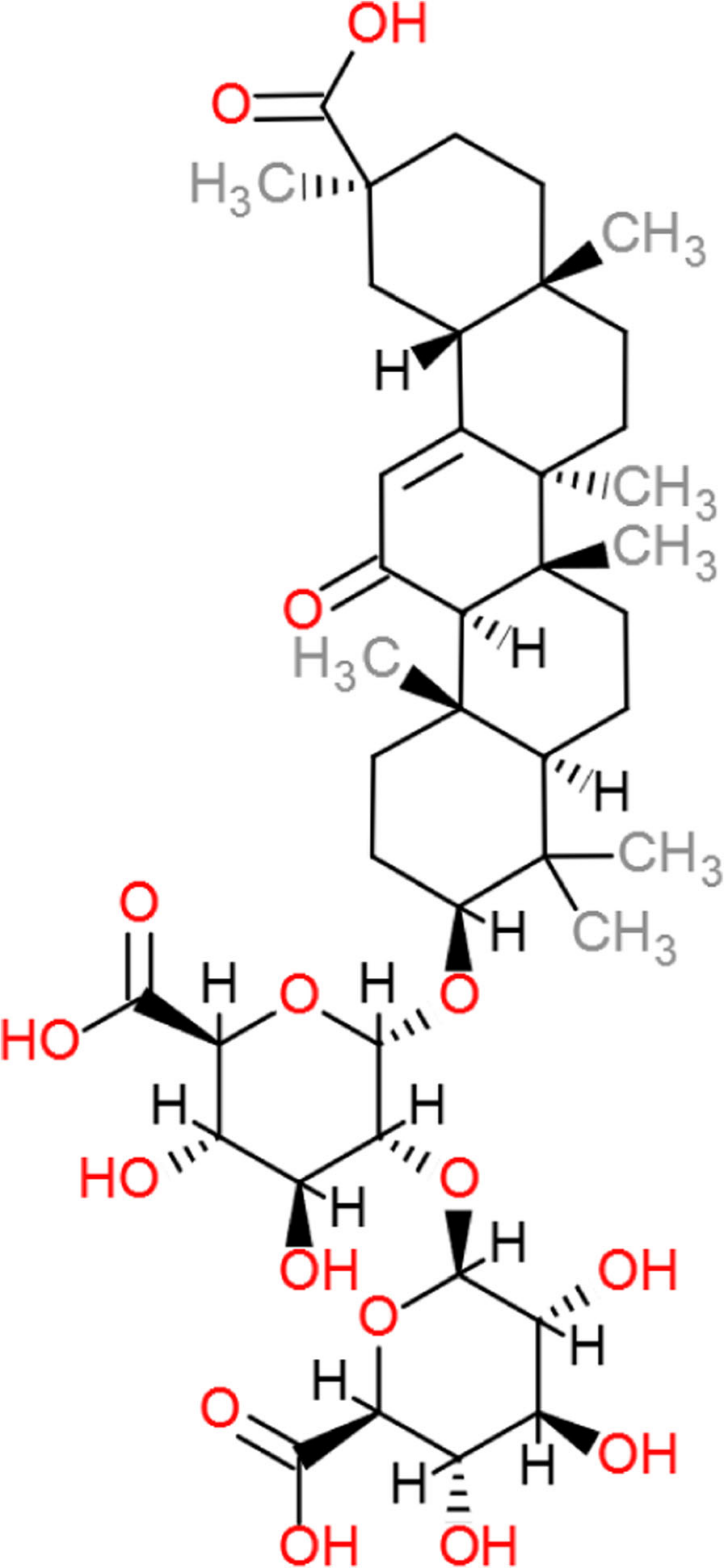
The chemical structure of licorice, *glycyrrhizin*. Modified after Li et al. [29]. Glycyrrhizin is the sweet component of licorice which is metabolized to glycyrrhetinic acid. Glycyrrhizin has potential to be anti-inflammatory. However, it also has the ability to cause retention of sodium and loss of potassium, increasing blood pressure, causing edema and affecting the renin-angiotensin-aldosterone system

Additionally, the high use of licorice among pregnant women in Denmark deserves further attention. Regular consumption of licorice – and hence the active compound glycyrrhizin (see Fig. 3) - inhibits the 11-β-hydroxysteroid dehydrogenase type 2 (11-β-HSD2) enzyme, thereby activating cortisol and generating hypokalaemic hypertension [21]. Notably, we observed a minor increase in maternal systolic blood pressure (*p* = 0.04) and lower birthweights among children exposed to frequent maternal intake of licorice. The downregulation of 11-β-HSD2 in the placenta may contribute to several of the pathways leading to an increased risk of preeclampsia [21], miscarriage [22], preterm birth [23], toxicological effects [24], and lower intelligence quotient, poor memory and increased risk of attention deficit in the child [25]. Furthermore, the phytoestrogen found in licorice, glabridin, might explain the pubertal advancement seen in girls prenatally exposed to licorice [25, 26].

In this study, we characterized lifestyle habits at the end of the first trimester of pregnancy, which is a very critical period of organogenesis [27]. Notably, several studies into the Developmental Origins of Health and Disease Hypothesis (DOHaD) have shown that different effect occur in responds to reprogramming at different part of the pregnancy (for a review see Roseboom and coworkers [28]). Further studies should be aimed at including additional information of the habits in later pregnancy and expand the number of participants as it cannot be excluded that changes in habits occur during pregnancy which could also affect offspring health.

## Conclusions

In a Danish context, ginger and liquorice are commonly ingested by pregnant women as are alternative medications. Based on our results and the discussion above, we recommend that health providers actively seek to increase their knowledge of the eating habits and alternative medicine use of pregnant women to avoid unnecessary health risk in pregnancy.

## Additional files

Additional file 1: **Table S2.** Overview of pregnancy outcomes at birth. (DOCX 13 kb)

Additional file 2: **Figure S1.** Flow diagram of participant involvement. (DOCX 24 kb)

Additional file 3: **Table S1.** Summary of the relationship between parity and maternal lifestyle among the pregnant women. (DOCX 13 kb)

## Abbreviations

BMI: Body Mass Index
DOHaD: Developmental Origins of Health and Disease Hypothesis
OTC: Over-The-Counter
UPLC-HR-TOFMS: Ultra-Performance Liquid Chromatography with High-Resolution Time-Of-Flight Mass Spectrometry Analysis
