# Supplementary material for: Use of alternative medicine, ginger and licorice among Danish pregnant women – a prospective cohort study
Source: BMC Complement Altern Med. 2019 Jan 5;19:5. doi: 10.1186/s12906-018-2419-y (PMC6320632; doi:10.1186/s12906-018-2419-y)
Supplement: Supplementary file 1 — Table S2. Overview of pregnancy outcomes at birth. (DOCX 13 kb) [file 12906_2018_2419_MOESM1_ESM.docx]

## Additional file 3. Table S3: Overview of pregnancy outcomes at birth.

| Pregnancy information | % (n) |
| --- | --- |
| Pregnancy |  |
| Singleton | 97.8(220) |
| Multiple pregnancy | 2.2(5) |
| Birth |  |
| Induction of labor | 61.3(46) |
| Labor augmentation | 38.7(29) |
| Delivery mode |  |
| Spontaneous vaginal delivery | 71.8(158) |
| Vaginal birth with vacuum extraction | 8.8(19) |
| Planned cesarean section | 5.1(11) |
| Emergency cesarean section | 13.4(29) |
| Antibiotics during birth | 8.3(18) |
| Postpartum bleeding (>500 ml) | 12.9(28) |
| Newborn health – Apgar score after 5 min. |  |
| 10 | 94.5(205) |
| 9 | 1.8(4) |
| 8 | 0.5(1) |
| 7 | 0.5(1) |
| 5 | 0.5(1) |
| 3 | 0.9(2) |
| 2 | 0.5(1) |
| 1 | 0.5(1) |
| 0 | 0.5(1) |
