# Supplementary material for: Use of alternative medicine, ginger and licorice among Danish pregnant women – a prospective cohort study
Source: BMC Complement Altern Med. 2019 Jan 5;19:5. doi: 10.1186/s12906-018-2419-y (PMC6320632; doi:10.1186/s12906-018-2419-y)
Supplement: Supplementary file 2 — Figure S1. Flow diagram of participant involvement. (DOCX 24 kb) [file 12906_2018_2419_MOESM2_ESM.docx]

Supplementary information

## Additional file 1. Figure S1: Flow diagram of participant involvement
