# Supplementary material for: Use of alternative medicine, ginger and licorice among Danish pregnant women – a prospective cohort study
Source: BMC Complement Altern Med. 2019 Jan 5;19:5. doi: 10.1186/s12906-018-2419-y (PMC6320632; doi:10.1186/s12906-018-2419-y)
Supplement: Supplementary file 3 — Table S1. Summary of the relationship between parity and maternal lifestyle among the pregnant women. (DOCX 13 kb) [file 12906_2018_2419_MOESM3_ESM.docx]

## Additional file 2. Table S1: Summary of the relationship between parity and maternal lifestyle among the pregnant women.

| Lifestyle | Nulliparous  % (n) | Primiparous  % (n) | Multiparous  % (n) |
| --- | --- | --- | --- |
| Maternal pre-pregnancy BMI |  |  |  |
| Underweight (<18,5) | 50.0(2) | 50.0(2) | 0.0(0) |
| Normal (18,5-24,9) | 41.9(39) | 49.5(46) | 8.6(8) |
| Overweight (25-30) | 35.8(24) | 47.8(32) | 16.4(11) |
| Obese (>30) | 46.7(28) | 33.3(20) | 20.0(12) |
| Maternal chronic disease |  |  |  |
| Yes | 48.4(31) | 43.8(28) | 7.8(5) |
| No | 38.8(62) | 45.0(72) | 16.25(26) |
| Civil status |  |  |  |
| Married | 22.3(21) | 55.3(52) | 22.3(21) |
| In relationship, cohabitating | 55.4(67) | 38.8(47) | 5.8(7) |
| In relationship, non-cohabitating | 60.0(3) | 20.0(1) | 20.0(1) |
| Single | 33.3(1) | 0.0(0) | 66.7(2) |
| Other | 100(1) | 0.0(0) | 0.0(0) |
| Highest education |  |  |  |
| Elementary school | 25.0(2) | 12.5(1) | 62.5(5) |
| Upper secondary school | 52.9(9) | 41.2(7) | 5.9(1) |
| Vocational education | 41.1(23) | 37.5(21) | 21.4(12) |
| Shorter level of education | 48.3(14) | 48.3(14) | 3.4(1) |
| Bachelor’s degree | 43.5(37) | 44.7(38) | 11.8(10) |
| Master’s degree | 24.1(7) | 66.7(18) | 7.4(2) |
| Other | 50.0(1) | 50.0(1) | 0.0(0) |
| Household income |  |  |  |
| <103,000 DKK | 50.0(1) | 0.0(0) | 50.0(1) |
| 103,000-200,000 DKK | 62.5(5) | 25.0(2) | 12.5(1) |
| 200,000-500,000 DKK | 40.5(32) | 45.6(36) | 13.9(11) |
| 500,000-800,000 DKK | 47.1(49) | 38.5(40) | 14.4(15) |
| >800,000 DKK | 19.4(6) | 71.0(22) | 9.7(3) |
|  |  |  |  |
| All information was self-reported. BMI information was included as pre-pregnancy BMI, and students were included based on their future highest educational level. |  |  |  |
